# Diagnostic Accuracy of 256-Detector Row Computed Tomography in Detection and Characterization of Incidental Pancreatic Cystic Lesions

**DOI:** 10.1155/2015/707546

**Published:** 2015-06-02

**Authors:** D. Ippolito, P. Allegranza, P. A. Bonaffini, C. Talei Franzesi, F. Leone, S. Sironi

**Affiliations:** Department of Diagnostic Radiology, School of Medicine, San Gerardo Hospital, University of Milano-Bicocca, Via Pergolesi 33, 20900 Monza, Italy

## Abstract

*Purpose*. To assess the diagnostic value of 256-detector row MDCT in the characterization of incidentally detected pancreatic cystic lesions (PCLs). *Materials and Methods*. We retrospectively reviewed 6389 studies performed on a 256-row detector scanner, wherein ≥1 PCLs were incidentally detected. Images from a total of 192 patients (99 females; age range 31–90 years) were analysed referring to morphologic predictive signs of malignancy, including multifocality, inner septa, wall thickening, and mural enhancing nodules. *Results*. We evaluated 292 PCLs in 192 patients (solitary in 145 and ≥2 in 47; incidence 2.05%). Size ranged from 3 to 145 mm (mean 15 mm); body was the most common location (87/292; 29.8%). Intralesional septa were detected in 52/292 lesions (17.8%), wall thickening >2 mm in 13 (4.5%), enhancing wall and mural nodules in 15 (5.1%) and 12 (4.1%), respectively. Communication with ductal system was evident in 45 cases. The most common diagnoses, established by histology or imaging analysis, were IPMNs (about 86%), while serous cystic neoplasia (3.7%) and metastases (0.5%) were the less common. *Conclusion*. MDCT provides detailed features for characterization of PCLs, which are incidentally discovered with increased frequency due to the widespread use of cross-sectional imaging.

## 1. Introduction

Due to the improved spatial resolution of cross-sectional imaging and to its widespread use in the daily practice, unexpected cystic lesions of the pancreas (pancreatic incidentalomas) are increasingly being recognized [1–3] in clinical settings with no direct correlation with pancreas itself. Hence, they often represent a diagnostic and therapeutic challenge.

Pancreatic cysts encompass a wide spectrum of benign, malignant, and borderline lesions that can either be primarily cystic or result from cystic degeneration of solid tumors [3–6]. They mainly include pseudocysts, mucinous, serous, and intraductal papillary mucinous neoplasms (IPMNs), cystic endocrine tumors, and, less frequently, metastases [7]. Poor information is currently available concerning their natural history. Although the risk of malignancy is considered higher in symptomatic lesions [3, 8], the majority of asymptomatic cysts are neoplasms, including IPMNs and mucinous cystadenomas [5]. Moreover, limited and discording data have been previously reported about the incidence and the prevalence (from 0.7% up to 36.7%) of incidentally detected pancreatic cysts on cross-sectional imaging [3, 8, 9].

No current imaging criteria exist to accurately distinguish between benign and malignant lesions [8]. However, the presence of some worrisome features (diameter ≥3 cm, thickened walls, enhancing mural nodules, main pancreatic duct/MPD size of ≥5 mm, and abrupt change in MPD caliber with distal pancreatic atrophy) has been correlated with an increased risk of malignancy [3, 10, 11]. Therefore, an accurate identification of these suspicious features is mandatory for appropriate lesions characterization and management, even in terms of follow-up [7, 11–13]. Multidetector computed tomography (MDCT) is referred to as the first-line imaging technique for investigation of main pathologic conditions involving pancreas. MDCT is also applied in the clinical practice in several management algorithms for cystic lesions because of its high spatial resolution and wide availability [12]. However, to the best of our knowledge there are no data published in the literature concerning the diagnostic accuracy of 256-detector row MDCT scanners in patients with pancreatic incidentalomas.

The purpose of our study was to evaluate the role and the diagnostic accuracy of 256-detector row MDCT for the depiction and the characterization of incidentally detected pancreatic cysts lesions (PCLs). Further, our second aim was to report clinical data about cystic lesions in terms of incidence, sizes, and morphological features correlated with a high risk of malignancy.

## 2. Materials and Methods

### 2.1. Study Population

A total of 6389 abdominal MDCT examinations performed in our department between January 2011 and February 2013 for different clinical purposes (i.e., oncologic staging and follow-up, CT angiography, or urography) were retrospectively reviewed. For final imaging analysis, only patients with incidental pancreatic cystic lesions and with the following criteria were included: (a) no evidence of pancreatic cystic lesions on previous available imaging studies, such as abdominal ultrasound (US) or Magnetic Resonance Imaging (MRI); (b) no history of pancreatic disease and/or previous pancreatic surgery; (c) no symptoms that could be referred to a pancreatic disease or dysfunction; (d) no proven Von Hippel-Lindau (VHL) or other syndromes correlated with risk of pancreatic involvement. MDCT studies where pancreas was not entirely covered in at least one of acquisition phases (either unenhanced or after contrast), those performed without contrast medium injection (i.e., because contraindicated), and those limited by patient motion or major image artifacts (preventing an appropriate evaluation of pancreas) were excluded. All the patients evaluated gave their informed consent for the MDCT study, after the explanation of the whole procedure.

### 2.2. MDCT Protocols

All the MDCT examinations were performed on a 256-slice scanner (Brilliance iCT, Philips Medical Systems, Best, Netherlands) and the different protocols employed, in terms of scanning parameters and number of dynamic phases acquired, varied according to the underlying clinical indications (i.e., tumor staging or follow-up and undefined abdominal pain).

Tube voltage ranges from 100 to 140 kV while tube current (mAs) is automatically determined by *x*-, *y*-, and *z*-axis dose modulation, ranging from 90 to 350 mAs. Slice thickness ranges from 2 to 5 mm. The dynamic study is performed after the intravenous injection of an iodinate contrast medium (Xenetix 350; Guerbet, Aulnay, France), using an automated injector. Two standard protocols are generally employed in our institution:

1. The first examination, mainly for oncologic staging purposes, encompasses an unenhanced acquisition followed by a triphasic study using bolus-tracking (B-T) technique (threshold of 100–120 HU): arterial (acquisition delay ranging from 7 to 13 seconds after the bolus, generally covering upper abdomen), venous (60–90 seconds), and equilibrium-delayed phase (150–180 seconds).
2. In patients undergoing standard follow-up (i.e., lung cancer), a single portal venous phase (80–90 seconds after injection) is acquired; arterial phase is additionally performed in cases of primary hypervascular tumors (i.e., renal cancer), to better rule out the presence of distant metastases.

### 2.3. Image Analysis

All images were reviewed on a PACS (IMPAX 6.4, Agfa HealthCare NV, Mortsel, Belgium), assuming as cystic an oval or round pancreatic lesion with a predominant or uniform low attenuation appearance. The detection of PCLs and the analysis of their morphologic features were performed evaluating axial images along with the multiplanar reformatted (MPR) ones. Curved MPR along the main pancreatic duct (MPD) axis were performed on a dedicated workstation (Brilliance Portal Workspace, Philips Medical Systems, Best, Netherlands), if not previously available.

For each incidentally detected PCL, location within the pancreas (head, uncinate process, body, and tail) was reported. Lesions' number (1 or =2) was also recorded. If more than one cyst was present, we recorded data of all the cysts detected in that single case. Standardized morphologic features and predictive signs of malignancy, as listed by the International Consensus Guidelines for the Management of MCN and IPMN of the Pancreas (2012) [11], were then evaluated: major lesion size (>2 cm, measured either on axial or MPR images), communication with MPD, MPD size (>5 mm), presence of inner septa, wall thickening (>2 mm), and mural enhancing nodules (Table 1). Patients' sex, age, and concomitant available clinical history were also recorded.

**Figure 1:**
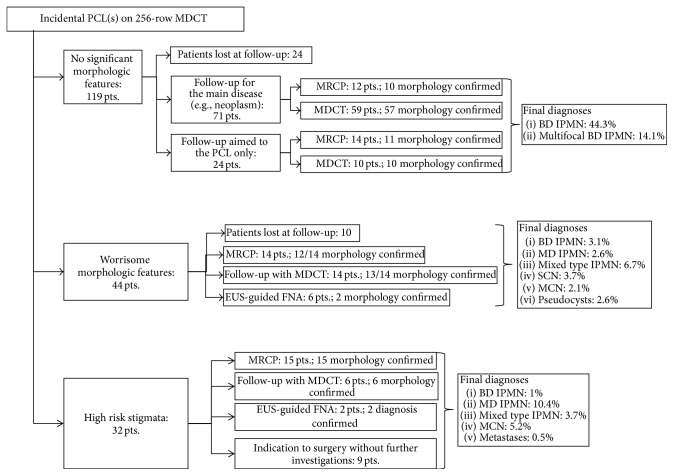
Correlation of MDCT imaging findings with further imaging follow-up studies (MRCP or MDCT), EUS-guided FNA data, or surgical specimen; final diagnoses are also reported for each group (patients stratified according to International Consensus Guidelines for the Management of MCN and IPMN of the Pancreas, 11). For overall final diagnoses (i.e., total IPMNs or MCNs) please refer to data reported in Table 3. MDCT: multidetector computed tomography; MRCP: magnetic resonance cholangiopancreatography; EUS: endoscopic ultrasound; FNA: fine-needle aspiration; IPMN: intraductal papillary mucinous neoplasm (BD: branch duct; MN: main duct); SCN: serous cystic neoplasm; MCN: mucinous cystic neoplasm.

On the basis of morphological features depicted on MDCT and according to the above-mentioned International Consensus Guidelines [11], patients were stratified in three different groups: “no significant morphologic features,” “worrisome features,” and “high risk stigmata.” Then we correlated MDCT findings with further imaging follow-up studied (either magnetic resonance cholangiopancreatography/MRCP or MDCT) or with EUS-guided FNA and surgical data whenever available (Figure 1).

### 2.4. Statistical Analysis

Clinical data of patients and morphological imaging features of lesions were recorded in an electronic datasheet. Patients' age and lesions' dimensions were reported as mean ± standard deviation (SD) and range while the evaluated morphologic MDCT features were reported as percentages.

## 3. Results

According to our previously described inclusion criteria, a total of 192 patients (99 females and 93 males) were retrospectively evaluated in our final analysis. Age ranged between 31 and 90 years, with a mean of 63 years (SD: 11 years) and a prevalence (162/192, 84%) of patients aged over 60 (Table 2). Among these 192 patients, a total of 292 unexpected pancreatic cysts were found, with an overall incidence of 2.05%. The main indications for MDCT were neoplasm staging or oncologic follow-up, urinary tract stones detection, and abdominal aortic aneurysms evaluation.

Lesions were homogeneously located within the gland as follows: head 81/292 (27.7%), uncinate process 47/292 (26.2%), body 87/292 (29.8%), and tail 77/292 (26.3%). Cysts' sizes ranged between 4 and 145 mm (mean 15 mm, SD: 15 mm). No correlation was observed between lesions' size and their location in the pancreas or patients' age. The majority of patients (145/192, 75.5%) had a single lesion while the remaining 47 cases demonstrated 2 (27/192, 14.1%) or even more (up to 8) lesions (20/192, 10.4%).

Concerning the morphologic features of PCL related to a higher risk of malignancy (Table 1), the one most commonly detected was the presence of inner septa (52/292 lesions, 17.8%). Communication with ductal system was observed in 25/292 (8.5%). Wall thickening >2 mm was present in 19/292 lesions (6.5%), presence of mural enhancing nodules in 12/292 (4.1%), and a concomitant dilation of MPD in 10/292 (3.4%).

**Figure 2:**
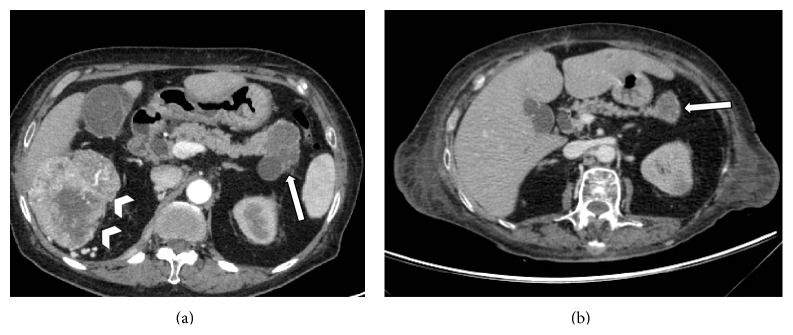
Axial MDCT images performed in a 72-year-old woman presenting with hematuria. At baseline MDCT arterious phase scan (a) demonstrates the presence of a primary neoplastic lesion of the right kidney (arrowheads) and a multiloculated hypodense lesion in pancreatic tail (white arrow), suspicious either for cistoadenoma or metastasis. At subsequent MDCT study performed after right nephrectomy and chemotherapy (b), the pancreatic lesion (white arrow) shows partial shrinkage, confirming the diagnosis of metastasis from renal neoplasia.

**Figure 3:**
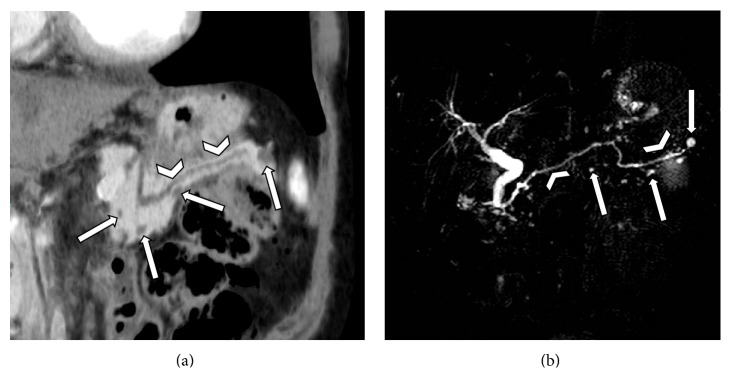
(a) MDCT study performed in a 65-year-old man referring to evaluation of hepatic disease. MPR curved reconstruction shows the main pancreatic duct (MPD) along its whole length (arrowheads) and several small cystic lesions (white arrows), without inner septa and no detectable connection with ductal system. These findings were considered consistent with multifocal branch type IPMN. (b) Radial images from a subsequent MRCP study confirm the regular caliper of MPD (arrowheads) and the presence of a higher number of multiple small cysts (white arrows) that spread within the whole pancreatic gland; even if a clear communication with MPD was not present, MRCP confirms the diagnosis of multifocal branch type IPMN.

The final diagnosis of the cystic lesions was established according to pathological findings, when available: images were correlated with histology in 41 patients who at last underwent surgery (21%) and in 25 cases (13%) with cytopathological findings obtained by fine-needle aspiration (FNA) under endoscopic ultrasound (EUS) examination. In the remaining 126 cases (66%) diagnosis was either based on images analysis or obtained by correlating MDCT findings with other imaging techniques (i.e., MRI and MR cholangiopancreatography/MRCP) or with follow-up studies (Figures 1–3). On these bases, the most common final diagnoses (about 86%) in our population study were branch type IPMNs (particularly single in 48.4% and multifocal in 14.1%); serous cystic neoplasia/SCN (3.7%), pseudocysts (2.6%), and metastases (0.5%) were the less common (Figure 1, Table 3).

## 4. Discussion

The increased employment of high-resolution cross-sectional imaging in the daily practice has raised the detection of several lesions, even malignant but often with an uncertain significance and frequently in clinical settings not directly correlated with the lesion itself. Thus, pancreatic cysts represent a common finding in asymptomatic patients undergoing MDCT or MRI studies. As such lesions include a wide spectrum of entities with different biological behaviours, the main task of imaging is to differentiate benign cysts (i.e., pseudocysts) from premalignant and malignant ones [1, 10, 14].

MDCT is a widely available technique with high spatial and temporal resolutions and is generally used as first-line examination for several diseases involving the pancreas, also including preoperative staging of malignancies [15]. MRI demonstrated better sensitivity than MDCT in depiction and characterization of pancreatic cysts [12] and with a reported higher frequency [11, 16], in particular for small lesions (<10 mm). There are also evidences that MRCP is superior in establishing diagnosis of IPMN, in determining their type and extent, and in distinguishing branch type from MCNs, because of higher accuracy in evaluating the ductal system [17]. Therefore, MRI is considered the gold standard radiologic imaging technique for PCL characterization [18]. The accuracy of endoscopic ultrasound (EUS) has been reported at least equivalent to MRI [19]. Moreover, even if invasive and strongly operator-dependent, EUS is feasible to obtain cytopathological and cystic fluid specimens by fine-needle aspiration biopsy (FNA) [20, 21].

The frequency of unexpected pancreatic cystic lesions has been the object of many studies. Data from autoptic series indicate that small cystic lesions (<10 mm) are present in up to 25% of cases [22]. In recent literature the frequency of pancreatic cystic lesions in MDCT routine examinations has been described but to our knowledge few papers [2, 3] evaluated patients undergoing MDCT without symptoms and/or history of pancreatic disease. This distinction is important in order to exclude patients whose cystic lesions could be correlated with pancreatopathy and, therefore, classified as “nonincidental.” Spinelli and colleagues [2] reported an incidence of 0.7% in 24.039 studies using 4-, 8-, and 16-row CT scanners, while Laffan et al. [3] found an overall incidence of 2.6% in 2382 CT examinations performed on a 16-row detector. Having an overall incidence of 2.05%, our study is in line with these data, raising the consideration that the employment of 256-detector row detector MDCT might not be strictly associated with a significant increase of depiction rate of unexpected small PCLs as compared to older scanners.

**Figure 4:**
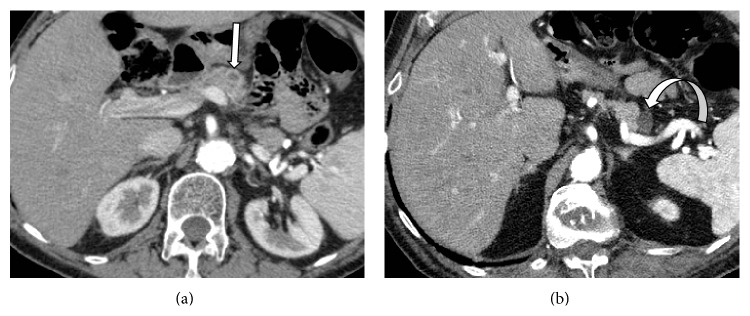
Two examples of pancreatic enhancement and cystic lesions' evidence in abdominal CT angiographic studies. (a) Poor contrast between the pancreatic parenchyma and cyst evident along the anterior edge of the gland (white arrow) and (78-year-old man) arterial phase does not allow proper lesion's evaluation (uncertain wall thickening). (b) Good contrast between pancreatic parenchyma and simple cyst (curved arrow), which demonstrates clear margins (66-year-old man).

It has been suggested that in patients with pancreatic lesions (either solid or cystic) MDCT should be performed with dedicated protocols, in order to increase its diagnostic yield [10, 13]. In our series all MDCT studies were not generally performed with a pancreas-tailored protocol, as we excluded patients with known pancreatic lesions or disease. This could theoretically lead both to an underestimation of the presence of unknown PCLs and to a reduced or erroneous depiction of important morphological features (i.e., mural thickening). For instance, arterious phases performed in angiographic studies with short scansion delay at B-T (7-8 seconds) may provide images with poor contrast between the pancreatic parenchyma and cystic lesions (Figure 4). Nevertheless, in our series the depiction of morphologic features correlated with a high risk of malignancy was generally feasible, in particular in lesions with diameter >1 cm. This can certainly impact on patients' management since, according to the International consensus guidelines [11], lesions with concerning morphologic features should be candidate to resection. Moreover, in such patients, other studies for further characterization may not change the clinical management and MDCT would offer a satisfactory overview of these lesions for the surgical approach [10, 11].

**Figure 5:**
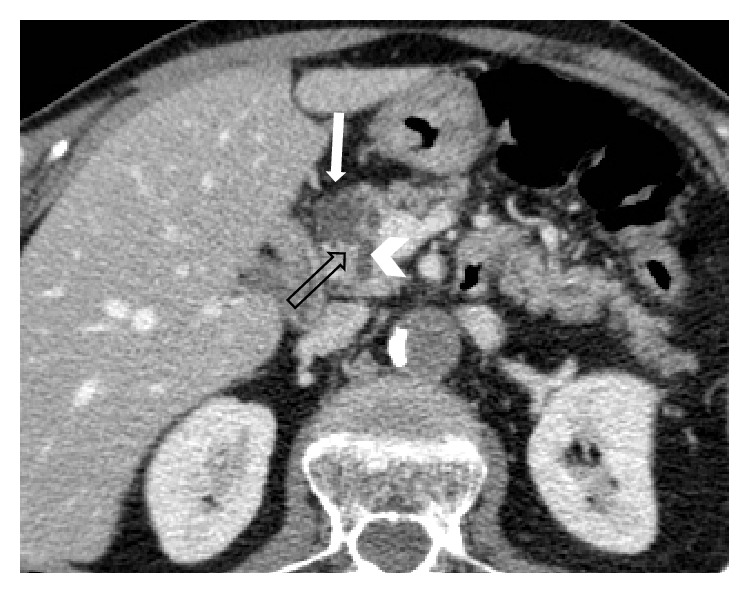
Axial MDCT scan acquired in the venous phase in a 58-year-old man referring to oncologic staging: a subtle branch duct (empty arrow) connects a 2 cm pancreatic cystic lesion (white arrow) of the head with the MPD (arrowhead).

**Figure 6:**
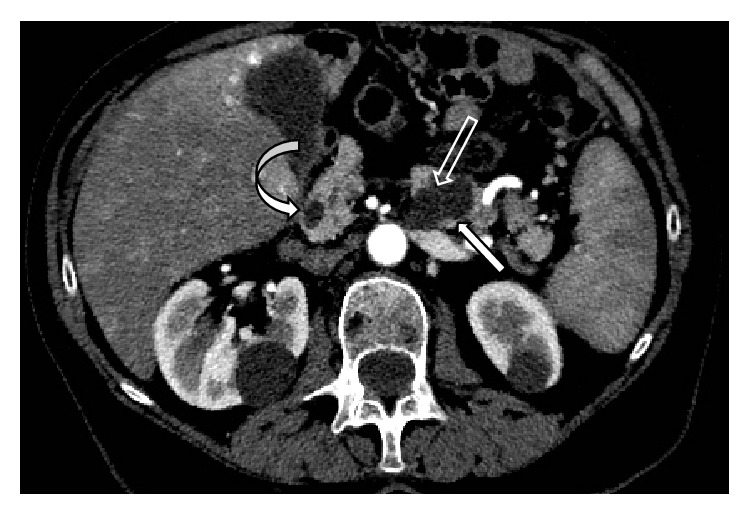
MDCT study performed on an 81-year-old woman for oncologic follow-up (no previous studies performed at our Institution). Axial arterial scan shows an unexpected cystic lesion (white arrow) in the body-tail of the pancreas. MDCT clearly demonstrates the presence of a subtle septum (empty arrow) within the cysts. A further concomitant simple cyst (curved arrow) is evident in the pancreatic head.

Even if it is proven that the presence of unsuspected <1 cm cystic lesions is underestimated in routine MDCT, as compared to MRI studies or autoptic series, small cystic lesions have reportedly a poor/scarce malignant potential [10] and even the cost-effectiveness of the follow-up of these lesions is controversial. In the present series none of the cystic lesions <1 cm has shown morphologic features associated with high risk of malignancy. Important features like nodules or septa could be seen more easily within larger lesions, having poor or no partial-volume artifacts and higher contrast between cystic fluid and solid structures. In terms of lesions characterization, the evidence of connection with the ductal system also represents a key-feature in distinguishing mucinous or serous cystic neoplasia from IPMNs. Our results indicate that, even not employing protocols tailored to imaging pancreas, a communication with main or branch duct can be properly detected by MDCT (Figure 5). When a clear communication between the lesion and the ductal system is not recognizable, a second line study (i.e., MRCP) might be employed to distinguish branch type IPMNs from mucinous cystic neoplasia (MCN) [11]. Presence of inner septa was the most common morphologic feature (17.8%) detected in our series of lesions (Figure 6). However, in some cases the reported presence of septa was the result of multiple adjacent cysts with thin wall rather than a single multiloculated PCL. Some previous studies [12, 13] reported that the presence of septa at MRI led to false positive results assuming histology as gold standard; this is probably due to changes in the structure of the lesion with subsequent rupture of septa over the time or during the histopathological processing. We found a concomitant dilation of main pancreatic duct (MPD) in only 3.4% of patients; among these cases, 70% (7/10) had cystic lesions with diameter >20 mm. This suggests that MDP dilation is a late consequence of the presence of pancreatic lesions producing an obstructing mass-effect on MPD.

In our study population, in the majority of cases patients underwent follow-up and/or second line studies. Even if a direct comparison among first MDCT scan and further imaging studies was not performed, we found that morphological features depicted on 256-detector row scanner were generally confirmed on subsequent studies. This was specifically evident in the most common instance: lesions with diameter <10 mm with no significant morphological features (single/multifocal branch type IPMN). This suggests that in such cases MDCT could be considered reliable for a proper characterization of these PCLs and even for their surveillance if required, particularly in patients already included in follow-up strategies for nonpancreatic disease, therefore sparing a redundant employment of MRCP. Therefore, MDCT even in presence of worrisome or high risk morphologic findings can have a significant role in patients with incidentally detected PCLs, helping clinicians in decision-making process.

This retrospective analysis presents some limitations. Studies were performed in different clinical settings and different protocols were used, varying from multiphasic studies to single venous phase examinations. Therefore, it is difficult to standardize the performance of 256-detector row MDCT in the evaluation of pancreatic cystic lesions. We did not assume a gold standard for our image analysis or even perform a direct comparison but we only analysed the available results of other radiological/pathological studies for the final diagnosis of our lesions, in order to determine the clinical value of MDCT in routine applications. Hence, no conclusive speculations can be done on accuracy of MDCT compared with other techniques.

Incidental pancreatic cystic lesions are discovered with increased frequency in clinical practice and a proper characterization is required to assess their malignant potential. The use of a 256-detector row CT scanner is apparently not associated with an increased detection rate of small (<1 cm) pancreatic cystic lesions, if compared with data from the literature obtained with old scanners. However, MDCT represents a powerful imaging technique for the depiction of morphological features related to a higher risk of malignancy, even when a pancreas-specific optimization protocol is not performed.

## Figures and Tables

**Table 1:** Analyzed morphologic features of cystic lesions, associated to a high risk of malignancy, according to International Consensus Guidelines for the Management of MCN and IPMN of the Pancreas, published in 2012 [11]. MPD: main pancreatic duct.

Main imaging features	Patients	Percentage
Inner septa	52/292	17.8%
Communication with ductal system (MPD)	25/292	8.5%
Wall thickening >2 mm	19/292	6.5%
Mural enhancing nodules	12/292	4.1%
MPD diameter >5 mm	10/292	3.4%

**Table 2:** Incidence of PCLs detected on MDCT studies, in relation to age ranges.

Age range (years)	Number of patients	Percentage
<40	5	2.6%
40–49	8	4.2%
50–59	14	7.3%
60–69	65	33.8%
70–79	69	36%
>80	31	16.1%
Total	**192**	

**Table 3:** Final diagnosis of the 292 pancreatic cystic lesions detected in 192 patients, according to histopathology, cytopahtological analysis, or imaging features evaluations. IPMN: intraductal papillary mucinous neoplasms.

Lesions		Percentage
IPMNs (85.9%)	Branch type	48.4%
	Multifocal branch type	14.1%
	Main type	13%
	Mixed type	10.4%

Cystadenomas (11%)	Mucinous	7.3%
	Serous	3.7%

Others (3.1%)	Pseudocysts	2.6%
	Metastases	0.5%
